# Combined proteomics/miRNomics of dendritic cell immunotherapy-treated glioblastoma patients as a screening for survival-associated factors

**DOI:** 10.1038/s41541-019-0149-x

**Published:** 2020-01-16

**Authors:** Friedrich Erhart, Matthias Hackl, Hannes Hahne, Johanna Buchroithner, Chen Meng, Simone Klingenbrunner, René Reitermaier, Katrin Fischhuber, Susanna Skalicky, Walter Berger, Sabine Spiegl-Kreinecker, Daniela Lötsch, Gerda Ricken, Bernhard Kuster, Adelheid Wöhrer, Georg Widhalm, Johannes Hainfellner, Thomas Felzmann, Alexander M. Dohnal, Christine Marosi, Carmen Visus

**Affiliations:** 1 Department of Neurosurgery, Medical University of Vienna, Vienna, Austria; 2 Institute of Neurology, Medical University of Vienna, Vienna, Austria; 3 Department of Tumor Immunology, St. Anna Kinderkrebsforschung Children’s Cancer Research Institute, Vienna, Austria; 4 TAmiRNA GesmbH, Vienna, Austria; 5 OmicScouts GmbH, Freising, Germany; 6 University Clinic for Neurosurgery, Kepler University Hospital, Johannes Kepler University, Linz, Austria; 7 Chair of Proteomics and Bioanalytics, Technical University of Munich, Freising, Germany; 8 Activartis Biotech GmbH, Vienna, Austria; 9 Institute for Cancer Research, Comprehensive Cancer Center, Medical University of Vienna, Vienna, Austria; 10 Research & Development, Central Blood Bank, Austrian Red Cross, Vienna, Austria; 11 Clinical Division of Medical Oncology, Medical University of Vienna, Vienna, Austria

**Keywords:** Cancer therapy, CNS cancer, Tumour immunology, Cell vaccines, Translational research

## Abstract

Glioblastoma is the most prevalent and aggressive brain cancer. With a median overall survival of ~15–20 months under standard therapy, novel treatment approaches are desperately needed. A recent phase II clinical trial with a personalized immunotherapy based on tumor lysate-charged dendritic cell (DC) vaccination, however, failed to prolong survival. Here, we investigated tumor tissue from trial patients to explore glioblastoma survival-related factors. We followed an innovative approach of combining mass spectrometry-based quantitative proteomics (*n* = 36) with microRNA sequencing plus RT-qPCR (*n* = 38). Protein quantification identified, e.g., huntingtin interacting protein 1 (HIP1), retinol-binding protein 1 (RBP1), ferritin heavy chain (FTH1) and focal adhesion kinase 2 (FAK2) as factor candidates correlated with a dismal prognosis. MicroRNA analysis identified miR-216b, miR-216a, miR-708 and let-7i as molecules potentially associated with favorable tissue characteristics as they were enriched in patients with a comparably longer survival. To illustrate the utility of integrated miRNomics and proteomics findings, focal adhesion was studied further as one example for a pathway of potential general interest.

Taken together, we here mapped possible drivers of glioblastoma outcome under immunotherapy in one of the largest DC vaccination tissue analysis cohorts so far—demonstrating usefulness and feasibility of combined proteomics/miRNomics approaches. Future research should investigate agents that sensitize glioblastoma to (immuno)therapy—potentially building on insights generated here.

## Glioblastoma: screening for survival-related factors

Glioblastoma is an aggressive form of brain cancer and effective immunotherapeutics are limited, with treatment currently based on chemotherapy and radiotherapy. A recent phase II clinical trial tested a personalized, targeted dendritic cell-based immunotherapy but there was no observed improvement in patient survival or progression-free survival compared to standard-of-care therapy. Here, Carmen Visus and colleagues have used tumor tissue samples from glioblastoma patients involved in this trial and receiving immunotherapy. Using a combination of mass spectrometry-based proteomics, microRNA sequencing and RT-qPCR they identified factors associated with survival or poor prognosis. Proteomics associated poor prognosis with various proteins including focal adhesion kinase 2 (FAK2), whilst microRNAs, miR-216b, miR-216a, miR-708 and let-7i, were associated with longer survival. Focussing on one pathway, FAK2, they integrated the proteomic and microRNA datasets and saw a negative association with overall survival across all patients. To test this, they added an FAK inhibitor to glioblastoma cell lines, including cells isolated from trial patients, and observed inhibition of gliomaspheres in treated cells, providing insights into potential immunotherapy targets.

## Introduction

Cancer immunotherapy is one of the recent breakthroughs in cancer treatment. For many malignant diseases, it has brought a remarkable improvement in outcomes.^1^ For glioblastoma—the most aggressive and most prevalent form of brain cancer^2^—various immunotherapeutic approaches have been tested but so far none of them has resulted in a real clinical breakthrough.^3,4^ Under the current therapeutic regimen of maximum safe resection, chemotherapy with temozolomide, radiotherapy (and where applicable additional modalities), a median overall survival of only 14.6–20.9 months is achievable.^5–7^ Innovative immunotherapeutic modalities under investigation in glioblastoma include immune checkpoint blockade, oncolytic viruses, chimeric antigen receptor (CAR) T cells and therapeutic vaccination.^8^ Immune checkpoint inhibitors block inhibitory immunoregulatory signaling loops such as CTLA-4 or PDL-1/PD-1 and led to remarkable survival improvements in cancers like melanoma.^9^ In glioblastoma, however, interim results from a phase III clinical trial were disappointing.^10^ Oncolytic viruses are designed not only to directly destroy glioblastoma cells but also to activate immunogenic cell death pathways leading to a stimulation of immune-responses. They are still in early clinical development phases.^8^ CAR T cells are bioengineered to harbor antigen recognition domains from antibodies connected to the activation domain from the T-cell receptor, which gives them the capability to precisely target defined tumor antigens. Early clinical data on glioblastoma indicated tumor infiltration and antigen-specific activity but no survival benefit^11^—possibly due to target antigen expression only in a fraction of all glioblastoma cells.^8^ Therapeutic vaccination includes peptide vaccines (e.g. rindopepimut,^12^ mutated IDH1^13^ and cellular vaccines—patient-derived dendritic cells (DCs) charged with defined peptide antigens (e.g. ICT-107^14^) or with whole tumor lysate.^15–17^ As of now, peptide-based vaccines (including peptide-charged DCs) did not lead to outcome improvements.^8^ In theory, using whole tumor lysate has the advantage of targeting multiple different tumor antigens at the same time.^8^ The whole tumor lysate DC vaccine “DCVax-L” has recently shown promising results in an interim analysis of a phase III clinical trial.^18^ But overall, a survival benefit has so far not been shown for DC vaccination against glioblastoma.

This is also true for the DC-based immunotherapy that we investigated in a phase II clinical trial (NCT01213407) of which the outcomes were recently analyzed (in October 2018).^19^ Seventy-six glioblastoma patients took part in it, 42 in the standard (control) therapy group and 34 in the DC immunotherapy group. The DC immunotherapy was a personalized, targeted technology given in addition to standard surgery plus chemoradiation: patients underwent leukocyte apheresis, DCs were generated in vitro from apheresis-derived monocytes, charged with autologous whole tumor tissue lysate, matured with lipopolysaccharides (LPS) plus interferon gamma (IFNγ) and finally injected into inguinal lymph nodes. Control patients received standard-of-care therapy without DC vaccination. No significant improvement of overall survival or progression-free survival could be reached^19^—despite evidence of tumor-directed immunostimulation triggered by the vaccine.^20^ Clinical parameters such as age, sex, O6-methylguanine–DNA methyltransferase (MGMT) methylation and isocitrate dehydrogenase (IDH) mutation did not have a measurable impact on survival in the immunotherapy cohort.^19^

On the one hand, the lack of efficacy in improving glioblastoma survival via DC vaccination was discouraging. But on the other hand, it posed an opportunity to study survival-associated factors and how they might inspire concepts for improving glioblastoma immunotherapy in the future. In the present paper, we describe the screening for molecules and pathways associated with clinical outcome. We used an innovative combination of two complementary investigation techniques on available tumor tissue samples: quantitative proteomics based on mass spectrometry and microRNA (miRNA) sequencing combined with RT-qPCR-based validation in an independent set of samples. For both methods, we identified differentially expressed factors between patients with a short-term (ST) survival versus patients with a long-term (LT) survival—based on the respective median values as typical cut-off.

We chose quantitative proteomics because it measures bona fide proteins and not only messenger RNA (mRNA) levels that not necessarily correlate with protein expression. And miRNA sequencing was selected since miRNAs are increasingly recognized as master regulators of tissue phenotypes.^21,22^ The proteomics part focused on factors associated with a dismal outcome (i.e. putatively supporting the “failure” of immunotherapy) and the miRNA part on LT survival-related factors that might skew glioblastoma tissue toward immunotherapy susceptibility.

As main findings, we established utility and feasibility of a combined proteomics/miRNomics approach and mapped potentially relevant survival-associated molecules. Based on that, we suggest a concept of combining (DC vaccination) immunotherapy and “tissue sensitizers”—e.g. small molecule inhibitors or miRNAs targeting multiple pathways—that might help to optimize treatment in the future.

## Results

### Proteomics feasibility: analysis of an ST and LT survivor confirms viability of protein quantification in available tumor cell samples

We started our analysis of potentially survival-related factors with an exploratory proteomics phase to establish feasibility of protein quantification in the single-cell suspension tumor samples that we had at our disposal. An arbitrarily selected ST and an LT survivor (based on the median) were analyzed by quantitative proteomics using a TMT10plex-based isobaric quantification approach.^23^ Three different types of sample were provided (lysates from tumor samples as well as two different types of single-cell suspension) and an initial total protein assay revealed that the protein amount of the provided samples was sufficient for comprehensive analyses (Fig. S1). Overall, 3287 proteins were identified, 2742 of them were quantified in both samples. High-abundance proteins like hemoglobin subunits and albumin naturally dominated the samples but also low-abundance proteins, such as endothelial growth factor receptor (EGFR) or calcium/calmodulin-dependent protein kinase type 1 (CAMK1), were quantifiable with high precision due to the isobaric labeling technology used. The network analysis depicted in Fig. S2 illustrates enriched pathways. For example, focal adhesion kinase 1 (FAK1) was upregulated in the ST survivor.

### Quantitative proteomics of 36 patients: unsupervised analysis reveals distinct survival-related subgroups in control patients

Next, we extended the initial exploration to an overall amount of 36 patient samples from the trial (20 control patients and 16 DC vaccination immunotherapy patients) measured in five TMT10plex sets. The selection of samples for this proteomics analysis followed the respective availability of tissue—as was the case in prior DC vaccination tissue studies by others.^16,24^ Given that the original clinical trial had been performed in multiple neurooncological centers all across Austria, technical factors like logistics, storage and quality control imposed limits on sample availability—leading to the subset of 36 samples out of initially 76 trial patients. No specific bias should have been introduced via that quasi-random sample set compilation mechanism. Still, to exclude any potential influence of factors with a known association with survival measures in glioblastoma, we investigated the impact of age, patient performance status (ECOG), MGMT methylation status and extent-of-resection. For none of these, a significant influence on survival could be registered (Fig. S3) in the immunotherapy patient set with tissue available for proteomics analysis. Thus, we deemed the available samples a viable set for proteomics studies aimed at mapping immunotherapy survival-associated factors.

In the hence following proteomics measurement, we identified 4713 proteins overall and 2477 of them were quantified in all 36 samples. Expectedly, the proteome profiles of the samples were relatively heterogeneous (not shown), and no batch effect of the normalized protein intensity from different TMT10plex experiments could be detected (Fig. S4). Also, the previous results from the initial exploratory protein quantification could be reproduced reliably (not shown).

To (generally) map sample subtypes defined by commonly overexpressed proteins, we performed *K-*means clustering of all samples (with 5 clusters; not shown) and extracted proteins overexpressed in these clusters by principle component analysis overlaying samples and proteins (Fig. S5). This approach revealed three distinct subtypes, one with a strong over-representation of synaptic and other neuronal proteins (cluster 3), oxygen transport and other blood proteins (cluster 1) and defense response proteins as well as antigen processing and presenting proteins (cluster 5).

**Fig. 1:**
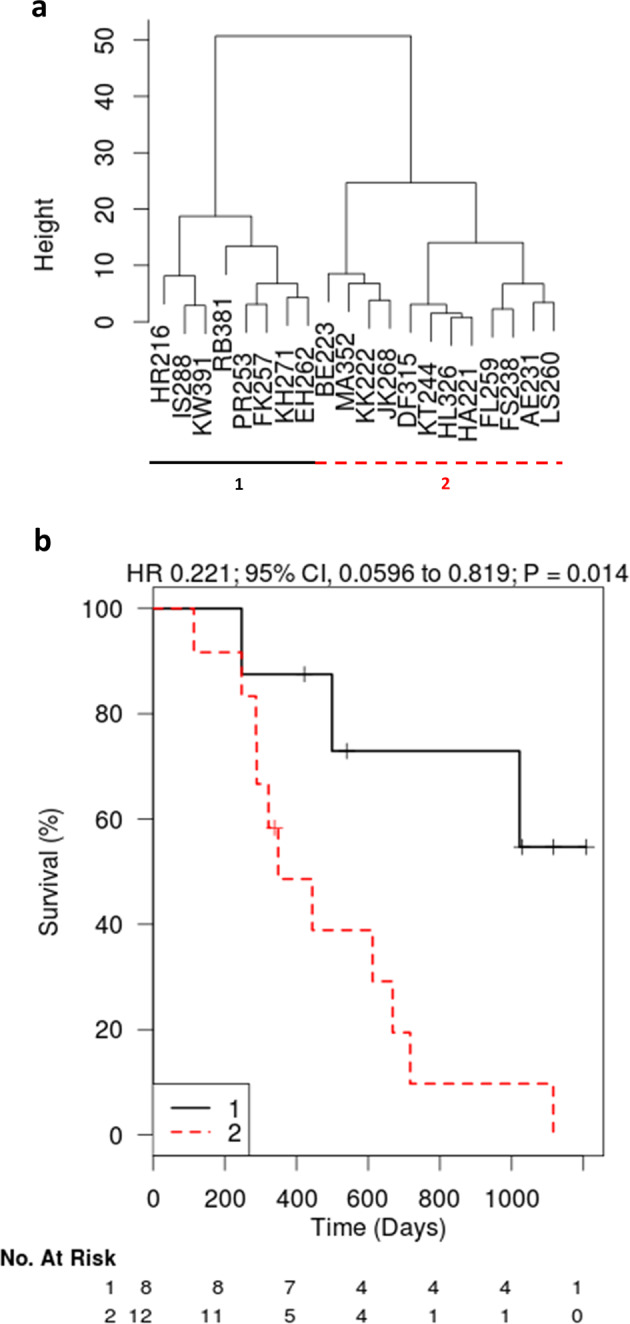
Proteomics-based identification of survival-relevant subgroups in (standard-of-care) control group patients (*n* = 20). “Height” represents an arbitrary unit for the similarity of the proteome profile of the respective samples (i.e. a measure of distance). The denominations at the bottom (e.g. HR216, IS288) represent single patients (pseudonyms). **a** Unsupervised clustering led to the definition of a group with a significantly worse survival outcome (*p* = 0.014) (**b**).

Following that, to identify survival-related subgroups of patients via analyzing the data from another angle, we performed an unsupervised hierarchical clustering. Here, the algorithm itself identifies survival subgroups (i.e. an ST- and an LT-surviving cohort) based on how the data clusters—as opposed to the other analyses where ST and LT were defined by us (see below). While for the (immunotherapy) treatment group this unsupervised approach did not produce significant results (after multiple testing correction), the (standard-of-care) control group samples segregated into two groups with significantly different overall survival when clustering the top three principal components (*p* = 0.014, log-rank test, Fig. 1). In total, 265 proteins were upregulated in the LT control group and 683 proteins were upregulated in the ST control group (Student’s *t* test, Benjamini–Hochberg corrected FDR < 5%) indicating massive differences of the molecular make-up of the two different subtypes. A network analysis based on functionally and physically interacting proteins using the String database^25^ revealed distinct strong networks and functional clusters of proteins associated with each subtype (Fig. S6). The LT subtype samples were characterized by relatively higher levels of oxidative stress-response and regulation of stress-response proteins, proteasome and ubiquitin-dependent proteolysis as well as carbohydrate and nucleotide metabolism. The ST subtype was dominated by proteins required for strong and sustained tumor cell growth such as proteins involved in protein biosynthesis (RNA splicing and processing, translation, aminoacyl tRNA synthetases and heat shock proteins), anti-apoptotic proteins, fatty acid metabolism and oxidative phosphorylation proteins as well as numerous protein kinases. Among the kinases potentially leading to sustained proliferative signaling and the evasion of growth suppression (Table S1) were protein kinase C (PKC), c-src tyrosine kinase (CSK) and mitogen-activated protein kinases 1, 3, 15 (MAPK1, MAPK3, MAPK15) as well as FAK1 and FAK2. They represent a set of candidate tumor drivers and/or factors likely connected to standard therapy resistance.

### Quantitative proteomics of 36 patients: consensus of supervised analysis and complementary techniques identifies survival-related factors in treatment patients

**Fig. 2:**
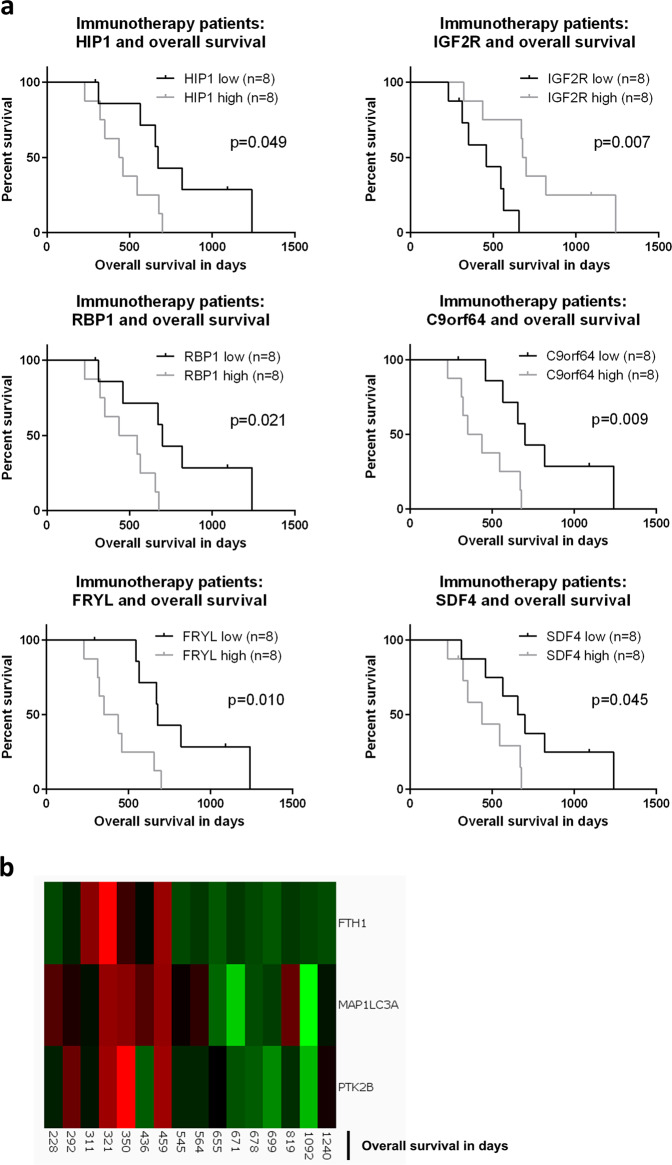
Identification of survival-relevant proteins in (immunotherapy) treatment group patients (*n* = 16). **a** The combination of supervised analysis, elastic net analysis and Kaplan–Meier testing identified survival-relevant proteins for immunotherapy patients. Kaplan–Meier curve analysis was based on stratification of patients with the median protein level as the cut-off. **b** Analyzing the data from a different angle, the combination of supervised analysis and Qlucore analysis led to proteins significantly enriched in ST immunotherapy patients. PTK2B = FAK.

Finally, to elucidate factors associated with ST or LT survival specifically in the immunotherapy treatment group, we performed a supervised analysis using a standard *t*-test to identify proteins highly expressed in ST or LT samples. To this end, tumor samples were again separated into an ST and an LT overall survival group based on the global median calculated from all patients, excluding censored patients with a shorter overall survival than median. In addition, we used an elastic net Cox model to identify survival markers which correlated significantly with overall survival. While the exploratory *t-*test resulted in 195 candidate markers (*p*-value < 0.05, no Benjamini–Hochberg correction), the elastic net approach identified 33 candidates associated with ST or LT overall survival. The consensus overlap of the two complementary approaches was defined as a set of potentially clinically relevant marker proteins (Table S2). A subsequent Kaplan–Meier analysis illustrated (Fig. 2a) that Huntingtin interacting protein 1 (HIP1), retinol binding protein 1 (RBP1) and chromosome 9 open reading frame 64 (C9orf64) were all associated with an unfavorable outcome in terms of overall survival (HIP1: *p* = 0.049, RBP1: *p* = 0.021, C9orf64: *p* = 0.009), while insulin-like growth factor receptor 2 (IGFR2) was significantly connected to a more favorable overall survival (Fig. 2a, *p* = 0.007). All other proteins from the consensus list did not reach statistical significance in the (univariate) Kaplan–Meier analysis. When cross-checking the proteins of interest (Fig. 2a) for an analogous impact in the control group patients, we observed that for HIP1 (*p* = 0.037) and IGF2R (*p* = 0.016), a survival-association was also present for control patients (Fig. S7). As opposed to that, for RBP1 (*p* = 0.059), C9orf64 (*p* = 0.735), FRYL (*p* = 0.623) and SDF4 (*p* = 0.711), no such relation was detected (Fig. S7)—they seemed immunotherapy-specific.

Lastly, to evaluate the bioinformatic results of the treatment group data from yet another angle, we combined the results of the previous analysis with the (multivariate) Qlucore Omics Explorer pipeline that identifies the most significant discriminating factors between groups.^26,27^ The top three factors enriched in ST immunotherapy survivors (Fig. 2b, *p* = 0.007) were ferritin heavy chain (FTH1), microtubule associated protein 1 light chain 3 alpha (MAP1LC3A) and protein tyrosine kinase 2 beta (PTK2B) that is also known as FAK2. Univariate Kaplan–Meier analysis, however, was not significant for these three proteins (FTH1: *p* = 0.074, MAP1LC3A: *p* = 0.067, FAK2: *p* = 0.202). The control group cross-check revealed a survival connection for FAK2 (as expected, see above, *p* = 0.003) but not for FTH1 (*p* = 0.912) and MAP1LC3A (*p* = 0.159).

Taken together, candidates possibly related to ST survival that we identified in the immunotherapy group were HIP1, RBP1, C9orf64, FTH1, MAP1LC3A and FAK2—even if the latter three were only detectable in multivariate analyses.

### Exploratory miRNA sequencing: analysis of ST and LT survivors in the treatment and the control group detects miR-216 as outcome influence factor

To complement the proteomics data, we analyzed miRNA transcription in tumor samples—with the goal of identifying potential master switches against immunotherapy failure factors, for miRNAs are increasingly seen as key regulators of cell and tissue states.^21,22^ One miRNA can regulate several hundreds of target mRNAs, thus largely determining the phenotype and function of a cell. As with the proteomics analysis, the set of available patient samples was a subset of all clinical trial patients. The tumor tissue source were formalin-fixed, paraffin-embedded (FFPE) samples. Again, the quasi-random selection was the result of external factors driving sample availability. Overall, a total of 38 samples could be retrieved, of them 18 from the immunotherapy group and 20 from the control group. Also here, to exclude any bias from age, patient performance status (ECOG), MGMT methylation or extent-of-resection, we tested for a potential impact of these variables in the available immunotherapy sample set. Like in the proteomics dataset, none of the variables had a survival-related effect in it (age: *p* = 0.426, ECOG: *p* = 0.104, MGMT: *p* = 0.420, extent-of-resection: *p* = 0.209, Fig. S8).

**Fig. 3:**
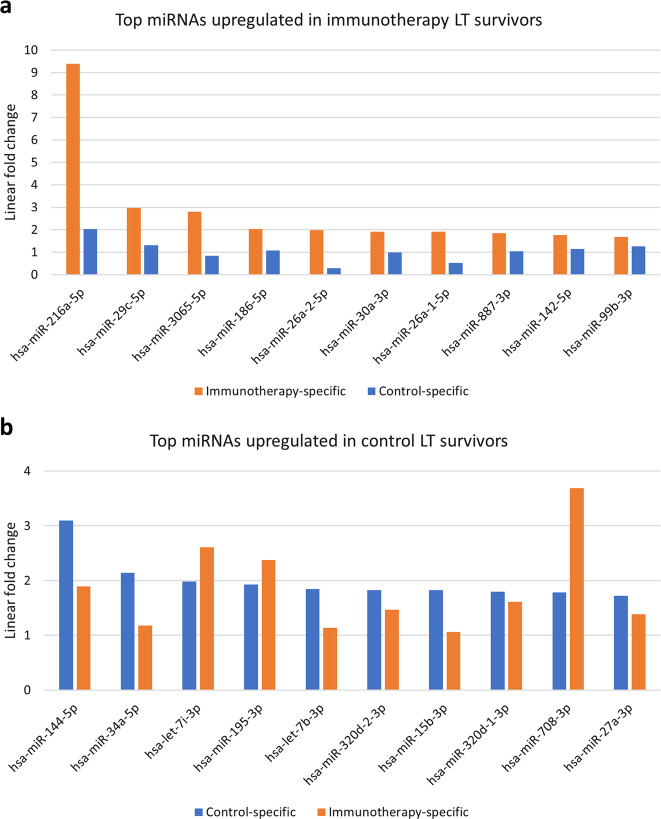
Results of miRNA sequencing in the exploration phase (*n* = 16 patients). Depicted are the top upregulated miRNAs in LT survivors of the immunotherapy treatment (**a**) or the standard-of-care control (**b**) group. **a** The miRNA with the highest upregulation in immunotherapy LT survivors was miR-216a-5p. **b** In the control group, it was miR-144–5p.

As a first step of the actual analysis, we performed small RNA sequencing of tumor tissue of immunotherapy and control group patients with survival times as extreme (short or long) as possible among the available specimens: 8 from the immunotherapy group (the 4 longest and the 4 shortest survivors in the set) and 8 from the control group (the 4 longest and the 4 shortest survivors in the set). This approach was designed to capture factors associated with valid biological function as opposed to statistical noise. Base quality, size distribution and GC content distribution indicated adequate sample quality (Fig. S9 shows an example). As a result, we identified 19 miRNAs differentially expressed in immunotherapy LT survivors versus ST survivors and 45 in standard therapy LT survivors versus ST survivors. Figure 3a, b give the top ten upregulated miRNAs in each treatment arm. MiR-216a was the top candidate for LT immunotherapy survivors. Interestingly, among the top ten miRNAs identified in the control group, we also found miRNAs—such as miR-708 and has-let-7i—that were even higher upregulated in the treatment group (Fig. 3b).

### Validation of exploratory results in 38 patients by RT-qPCR: The overall profile of tumor-specific, brain-specific and peritumour-specific miRNAs is the same across all samples

**Fig. 4:**
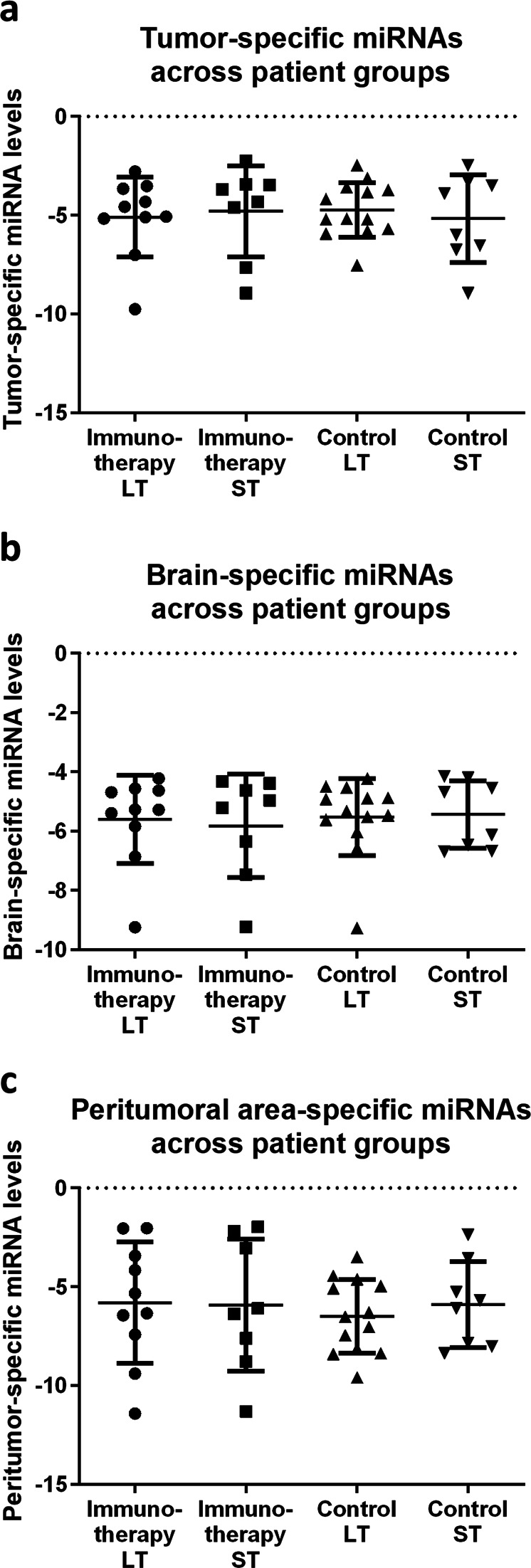
Evaluation of the FFPE material composition to rule out a potential bias. Comparison of the relative FFPE sample distribution based on miRNAs typical for the glioblastoma tumor itself (**a**), the healthy brain (**b**) or the peritumoral invasion area (**c**). The figure shows the average delta Cq-value for the respective tissue-specific miRNAs: three miRNAs specific for central glioblastoma tissue, five specific for the peritumoral invasion area and two specific for regular, healthy brain. **a** Across immunotherapy (treatment) and standard-of-care (control) LT and ST patients (*n* = 38), there is no significant difference in the abundance of tumor-specific miRNAs (*p* = 0.943). **b** The same is true for miRNAs specific for the “healthy” brain (*p* = 0.954). **c** Also for the peritumoral area, the distribution is the same across samples (*p* = 0.916). Error bars = standard error of the mean (SEM).

Next, we aimed at validating the miRNAs identified in the exploratory phase. For this purpose, we assembled a confirmatory panel of 58 miRNAs of interest based on the sequencing results (Table S3). This panel also included a set of control miRNAs from the literature: three miRNAs specific for the central glioblastoma tumor-tissue itself, five specific for the peritumoral invasion area as well as two specific for regular, “healthy” brain tissue.^28–31^ This total set of ten known miRNAs was used to control for potential tissue compartment heterogeneity in the 38 FFPE tumor sections (18 treatment, 20 control patients). The 58 miRNAs were quantified in 38 FFPE samples using RT-qPCR. It was observed that the abundance of tumor-specific, peritumoral area-specific or normal brain-specific miRNAs was not statistically different across the four experimental groups (Fig. 4, tumor: *p* = 0.943, peritumoral: *p* = 0.916, brain: *p* = 0.954). We concluded that the relative tissue composition of FFPE tumor samples was similar across the experimental groups.

### Validation of exploratory results in 38 patients by RT-qPCR: miR-216a, miR-216b, miR-708 and let-7i are associated with a more favorable outcome under immunotherapy

**Fig. 5:**
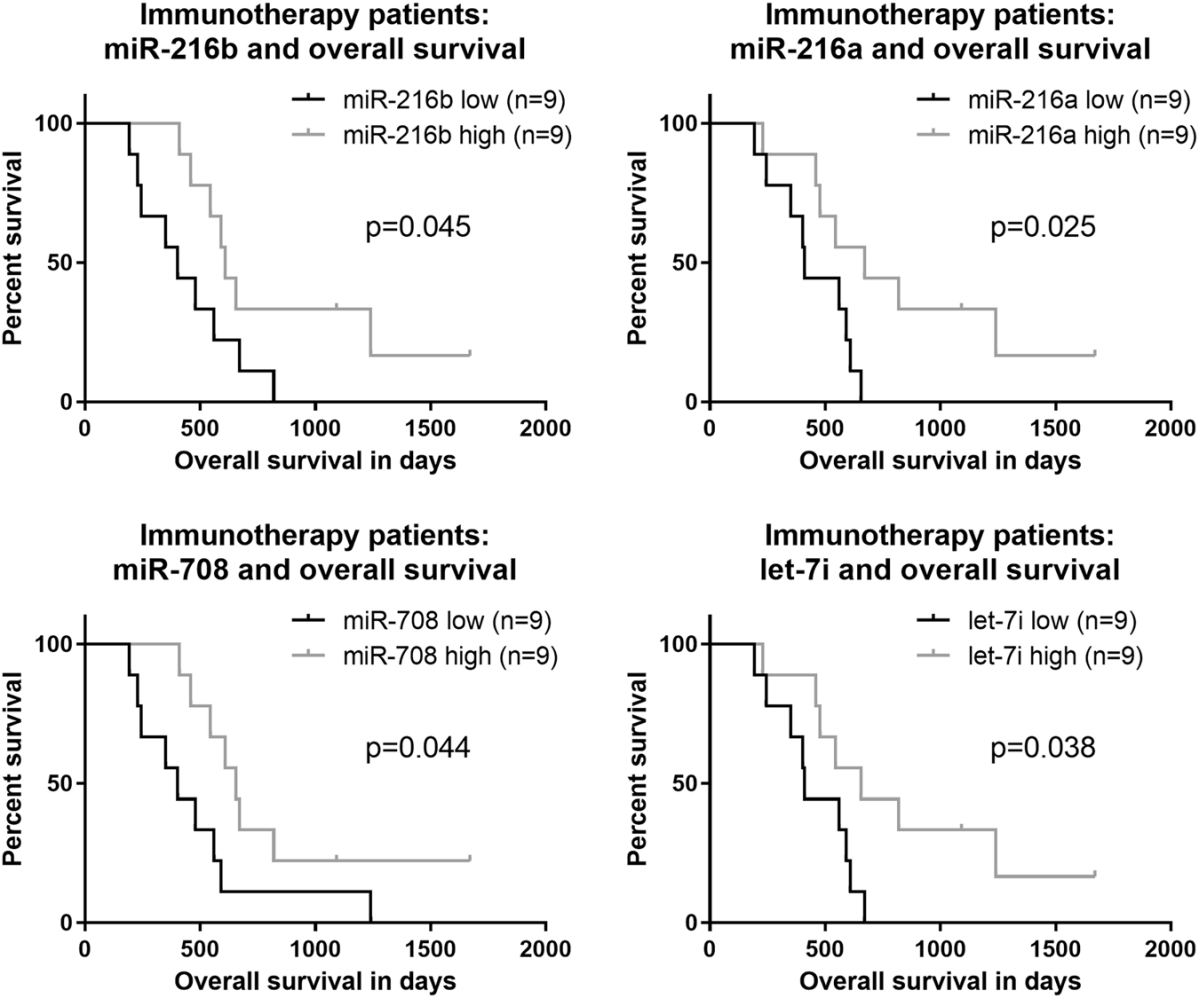
Results of miRNA qPCR measurement in the validation phase. Top four miRNAs identified in immunotherapy treatment patients (*n* = 18) with a relation to survival. Again, patient stratification for Kaplan–Meier curves was based on the median miRNA level as the cut-off.

Finally, when analyzing the miRNAs upregulated in LT immunotherapy survivors (as measured by qRT-PCR), we identified miR-216b, miR-216a, miR-708 and let-7i and confirmed an association with survival—they could significantly separate survival curves in the treatment group (Fig. 5, miR-216b: *p* = 0.045, miR-216a: *p* = 0.025, miR-708: *p* = 0.044, let-7i: *p* = 0.038). They are the four top candidates identified to potentially counteract unfavorable tissue factors in glioblastoma immunotherapy.

Subsequently, we aimed at understanding the biological pathways affected by these four miRNAs. Thus, we performed a KEGG target prediction^32^ for the four candidates miR-216b, miR-216a, miR-708 and let-7i. The results are shown in Table 1. Among the pathways likely regulated by the four miRNAs were the neurotrophin signaling pathway, focal adhesion and the transforming growth factor beta (TGFβ) signaling pathway.

**Table 1:** In silico target prediction for the top four miRNAs identified as survival-relevant via miRNA qPCR measurement in the validation phase.

Target prediction for miR-216a, miR-216b, miR-708 and let-7i		
KEGG pathway	*p*-Value	#Genes
Neurotrophin signaling pathway (hsa04722)	2.119459e−06	15
Focal adhesion (hsa04510)	5.546167e−06	20
TGF-beta signaling pathway (hsa04350)	6.549631e−05	9
Pathways in cancer (hsa05200)	0.0001311966	28
Protein processing in endoplasmic reticulum (hsa04141)	0.0003690133	15
MAPK signaling pathway (hsa04010)	0.0003891825	20
Cell cycle (hsa04110)	0.0003891825	11
PI3K-Akt signaling pathway (hsa04151)	0.0007148867	24
Colorectal cancer (hsa05210)	0.0007992389	8
Small cell lung cancer (hsa05222)	0.001927931	9

### Integration of proteomics and miRNA analysis: the focal adhesion pathway is explored as one select example

To illustrate the usefulness of the findings from our combined proteomics/miRNomics approach, we decided to perform an in-depth analysis of one pathway system identified as an example. For that, we chose the focal adhesion (kinase) pathway, because, when synthesizing the results from the proteomics and the miRNA analyses, it emerged consistently: FAK1 and FAK2 were among the proteins characteristic for standard treatment patients with a more dismal overall survival. Similarly, in the analysis of proteins related to immunotherapy failure, FAK2 was one of the identified factors. And miRNA target prediction indicated that miRNAs associated with a more favorable outcome downregulate the focal adhesion pathway—which again hints at focal adhesion as a potentially relevant factor. Summing up, these findings suggested that higher focal adhesion might be detrimental to the clinical outcome—without a definitive answer whether this would be specific or at least more relevant for immunotherapies.

**Fig. 6:**
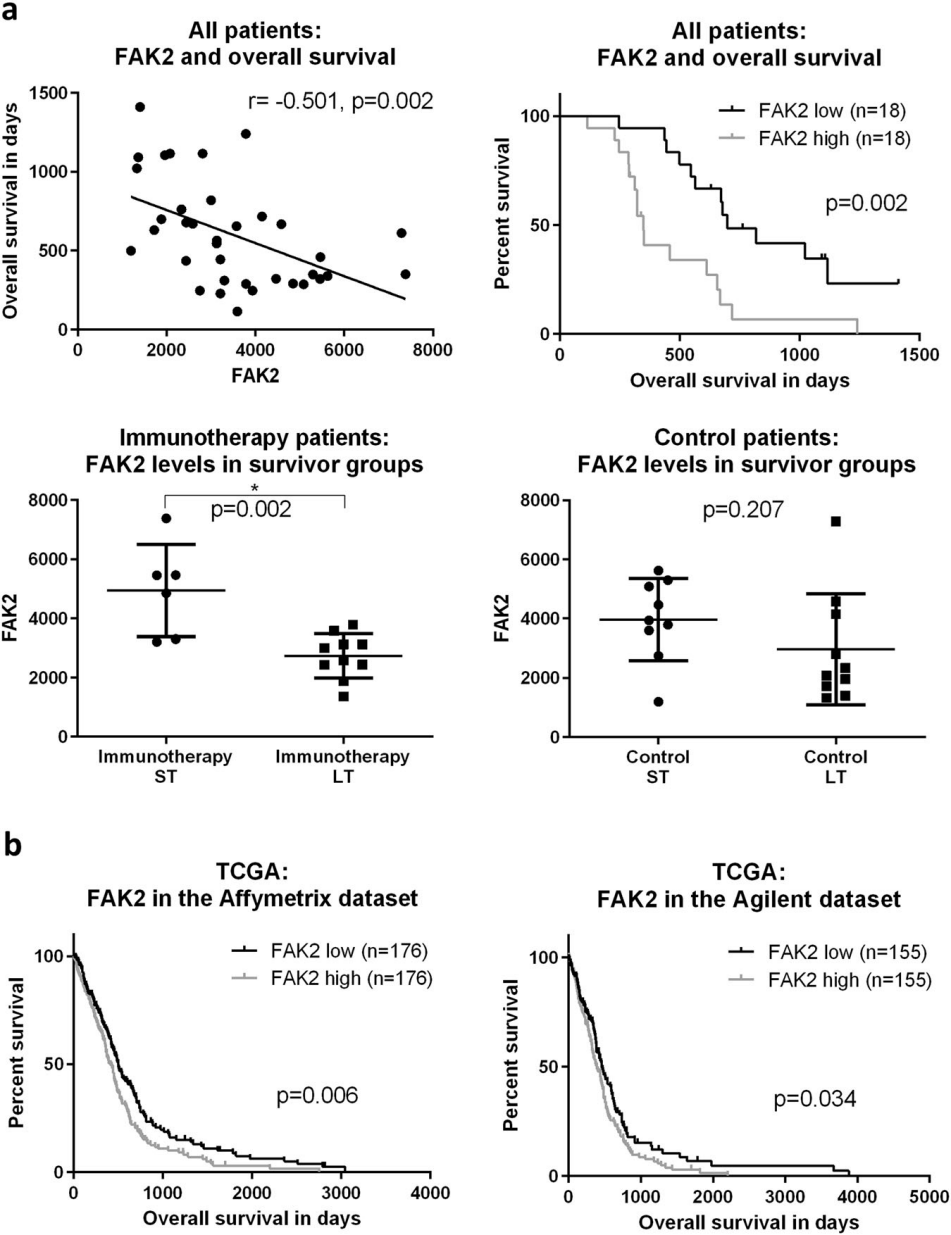
Possible role of focal adhesion for glioblastoma treatment failure. **a** Focal adhesion kinase 2 (FAK2) was measured in the quantitative proteomics analysis: across all proteomics patients (*n* = 36), it correlated negatively with survival (*p* = 0.002) and could significantly separate survival curves (*p* = 0.002). While it was significantly lower in treatment LT patients (*p* = 0.002), this was not the case for control patients (*p* = 0.207). **b** Data from the publicly available TCGA dataset confirm that patients with relatively high expression of FAK2 mRNA live significantly shorter (*n* = 352 and *n* = 310, respectively).

Therewith deducting a potential general interest in focal adhesion mechanisms in glioblastoma, we explored the focal adhesion system in our context further—with a focus on FAK2 (Fig. 6): Across all the patients measured (immunotherapy and control standard treatment), FAK2 had a significantly negative association with overall survival (*p* = 0.002). In line with that observation, in a Kaplan–Meier analysis, FAK2 levels could significantly separate survival curves—patients with “high” levels (above the median) lived significantly shorter (*p* = 0.002). When comparing immunotherapy-treated patients and standard (control) therapy patients, we observed that FAK2 levels were significantly higher in immunotherapy ST survivors than in immunotherapy LT survivors (*p* = 0.002). For ST standard therapy patients, this was not the case (*p* = 0.207). When contrasting miR-216b and FAK2, we registered a trend toward a negative correlation but without reaching statistical significance (Fig. S10, *p* = 0.434). To study FAK2 also in more extensive datasets than ours, we investigated gene expression and survival data from The Cancer Genome Atlas (TCGA). We selected the two largest available datasets (Harvard/MIT with Affymetrix measurement and University of North Carolina with Agilent measurement) and again defined groups based on FAK2 expression (Fig. 6b). Consistent with the data from our cohort, patients with “high” FAK2 levels lived significantly shorter in both datasets (Affymetrix: *p* = 0.006, 502 vs 414 days; Agilent: *p* = 0.034, 459 vs 393 days)—consistent with a potential general role for FAK2 in glioblastoma.

### FAK inhibition prevents sphere-formation of glioblastoma cells from trial patients

**Fig. 7:**
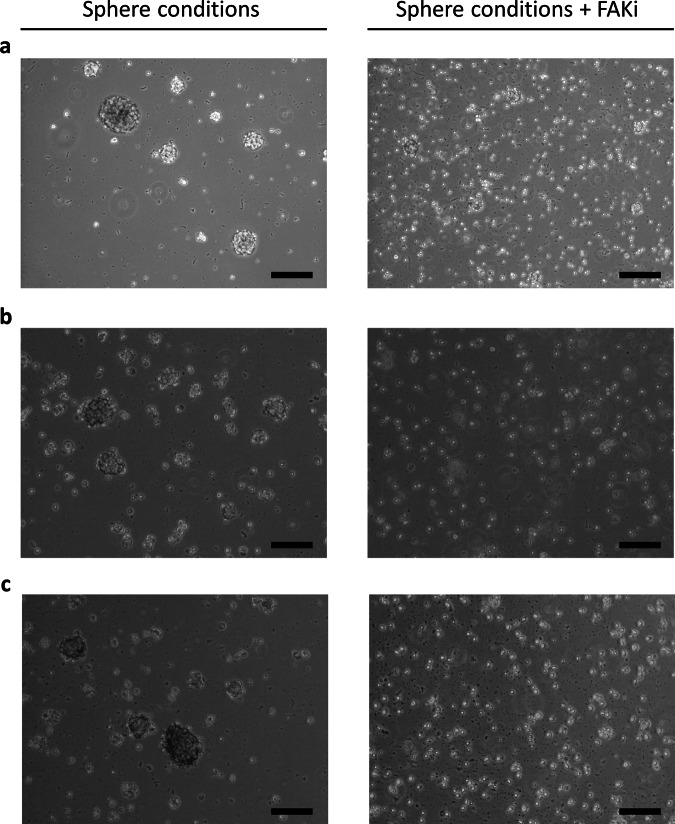
A focal adhesion kinase inhibitor can prohibit the formation of gliomaspheres. With a FAK inhibitor, glioblastoma cells stay in a state of single-cell suspension (right pictures) as opposed to without it (left pictures). Scale bars = 200 µm. **a** This holds true for the reference gliomasphere cell line NCH421K. **b** Gliomaspheres generated from the tumor tissue of a long-term surviving patient show the same behavior. **c** So do gliomaspheres of a cell line from a short-term surviving patient.

In some cancers, FAK inhibitors are already undergoing clinical trials.^33^ Thus, we were interested in the effects that FAK inhibition might have on glioblastoma cells from our trial patients (Fig. 7). We hypothesized an effect on the multicellular geometry of the tissue.

Hence, we complemented the previous data with first exploratory in vitro experiments. We focused on gliomaspheres—glioblastoma cells cultured in sphere-promoting, serum-free media—as their transcriptional profile resembles the original in vivo tumor better than adherent cell culture.^34^ Also, from a geometrical point of view, gliomaspheres represent the three-dimensional structure with the highest degree of intercellular adhesion. Therefore, we tested the effect of adding a FAK inhibitor (1,2,4,5-benzenetetraamine tetrahydrochloride) to glioblastoma cell lines while being in sphere-forming culture conditions. One line was NCH421K, a well-established gliomasphere cell line.^35,36^ Two other gliomasphere cultures had been generated from patients from the DC vaccination trial.^37^ One patient was an immunotherapy ST survivor, the other one an immunotherapy LT survivor. In all three cultures, addition of the FAK inhibitor (at a concentration of 10 µM, that only marginally affects cell viability^38^) prevented the formation of gliomaspheres (Fig. 7). Glioblastoma cells rather stayed in single-cell suspension. Apparently, FAK inhibition thus influenced the multicellular structure of glioblastomas in vitro in such a way that gliomaspheres were not formed. This is in line with previous experiments by others^39,40^ that performed analogous in vitro research—in a non-immunotherapy setting, though.

## Discussion

In the present work, we explored an innovative approach of combining quantitative proteomics and miRNA analysis for the identification of factors potentially associated with survival under DC vaccination immunotherapy in glioblastoma and what can be deducted for improving therapeutic approaches. Quantitative proteomics analysis identified candidate proteins (e.g. RBP1, FRYL, FTH1) connected to a more dismal outcome—among them FAK2 that was found for treatment as well as control patients. Complementary miRNA analysis explored molecules related to counteracting (immunotherapeutic) treatment failure. In our specific immunotherapy context, we identified miR-216b, miR-216a, miR-708 and let-7i as molecules of interest. Target prediction indicated that they for instance regulate the focal adhesion pathway. As an illustration of potentially insightful deductions from combined proteome/miRNome measurements, we examined focal adhesion further. In the two largest TCGA glioblastoma datasets, patients with “high” FAK2 abundance fared worse than others, which speaks for a general role of FAK also independent of DC vaccination. In vitro, FAK inhibitors prevented the formation of multicellular adhesion in the form of gliomaspheres. Taken together, focal adhesion mechanisms might be regarded as one pathway of overall interest for future research—possibly for DC vaccination and/or standard therapy.

In general, the research performed by us has important limitations and at the same time considerable strengths. In terms of caveats, the data stem from a limited number of patients (*n* = 36 for quantitative proteomics and *n* = 38 for miRNA analysis) from just one immunotherapy trial. Furthermore, even though we consider the results as generally relevant for all glioblastoma immunotherapies at large, this study had only one type of cellular immunotherapy in it (DC vaccination). It is not clear whether the findings are directly transferable to small molecule immunotherapies like checkpoint inhibitors. Further research in larger groups of patients with a diverse set of immunotherapies will be necessary to clarify. Nonetheless, we assume at least partial applicability to other immunotherapies: even though different immunotherapies vary in the exact mechanism, they still all share the common goal of immunostimulation. Secondly, the specificity of our findings for immunotherapies is not unanimously established. For some of the proteins identified when analyzing the DC vaccination cohort, we also registered a survival-relevance in the control group (e.g. FAK2, HIP1, IGF2R). This might indicate an effect that is so universal that it impacts multiple treatment modalities. Or, it might signal limitations inherent to the dataset. Future research will shed more light on this. Until then, the identified factors should be seen as what they are: survival-factor candidates from an initial screening.

A third consideration to be made regarding the present work is that so far mechanistic miRNA studies have not been performed by us. We anticipate focal adhesion regulation of the miRNAs identified but confirmatory cell culture experiments will have to be carried out as a next step. For that, e.g. miR-216b could be overexpressed in vitro in glioblastoma cell lines and mRNA sequencing could validate the respective downregulation of molecules from the focal adhesion pathway.

In terms of strengths, we see three main positive aspects in our work: First, we are adding a fresh perspective to a rapidly evolving therapeutic field in a disease area where insights into therapy deficiency are desperately needed. Glioblastoma is still one of the deadliest cancers and as of now the promise of immunotherapy could not reach a breakthrough. Also, there are only very few publications that investigate immunotherapy failure factors in glioblastoma material from a clinical immunotherapy trial.^41^ Second, we successfully combined two innovative, broad research methods that comprehensively characterize tissue states. To the best of our knowledge, we are so far the only ones to specifically use mass-spectrometry-based proteomics plus miRNA sequencing on material from a DC glioblastoma immunotherapy trial. Overall, our DC vaccination patient cohort is one of the largest so far in terms of glioblastoma tissue analysis.^42,43^ The fact that both complementary methods hinted at the same biological mechanism is noteworthy and underscores its potential clinical relevance. Our findings also validate that miRNAs can reliably be measured in glioblastoma FFPE samples stemming from a clinical context. While mRNAs are typically degraded in FFPE samples, the much shorter miRNAs seem to be largely conserved. Third, we pave the way for future research into novel combination therapies. We suggest that “tissue sensitizers” should be investigated that prepare the ground for immunotherapy via sensitizing glioblastoma tissue to it. For instance, FTH1 as a survival-associated factor candidate hints at the need to disrupt iron trafficking in glioblastoma, which fits prior work by others.^44^ More importantly, if confirmatory studies verify a role of focal adhesion, it might make sense to combine immunotherapy, and/or standard therapy, with agents that inhibit it. Interestingly, it has recently been shown that FAK inhibition and immunotherapy can be synergistically effective in pancreatic cancer.^45^ And in squamous cell carcinoma, it has been discovered that FAK promotes tumor evasion by inducing an immunosuppressive microenvironment, recruiting regulatory T cells and inhibiting cytotoxic CD8+ T cells.^46^ All this might hold true for glioblastoma, too.

When speculating about hypothetical future clinical concepts, FAK inhibition in addition to glioblastoma therapy could either be achieved via small molecule FAK inhibitors—such as the one we used—or miRNAs. The advantage of small molecule FAK inhibitors is that they are already in clinical investigation for a number of malignant diseases.^33,47^ Interestingly, a recent chemical proteomics study identified 15 different protein kinase inhibitors already in clinical trials which also target FAK2 with sub-micromolar potency^48^—most of them are not FAK2-selective, though. In glioblastoma, FAK inhibitors have so far only rarely been used in clinical trials and that mainly in the general context of solid tumors.^49^ There are some in vitro studies that test the effect of FAK inhibitors on glioblastoma cells. They typically find altered adhesion properties.^39,50,51^ So far, direct clinical data were rare. We contribute to closing that gap as the present study gives first early arguments for further pursuing the development of FAK inhibitors toward a potential future clinical application—especially as putative “sensitizers” for immunotherapy.

Alternatively, the advantage of developing *miRNA*-based combination therapies is that miRNAs not only interact with one single pathway but target multiple of them. For instance, our in silico prediction also identified the TGFβ pathway and the MAPK pathway. Both have been intensively discussed as therapeutic targets in many cancers—including glioblastoma.^52–55^ TGFβ inhibition, for example, has been described as a method to target stem-like cells in glioblastoma.^56,57^ Likewise, genomic alterations inducing constitutive activation of MAPK have been linked to master regulators of stemness in glioblastoma.^58^ Interestingly, also FAK inhibition has been established in vitro as a strategy to approach glioblastoma stem-like cells.^59^ Harnessing the potential of miRNAs, such as miR-216, could have a broader impact than conventional small molecule inhibitors. Until now, miR-216 has mainly been implicated in pancreatic cancer^60^ and nasopharyngeal cancer^61^ but rarely in glioblastoma.^62^ To the best of our knowledge, a connection to glioblastoma immunotherapy has not been made yet. As so far no miRNAs are in routine clinical usage in any disease, the effort for developing miRNA-based therapies, though, will be considerably higher than for small molecule FAK inhibitors.

When it comes to the potential biological meaning of FAK inhibition in the context of glioblastoma therapy, we speculate about two main mechanisms. Our in vitro experiments are preliminary but add an important perspective. First, if the adhesion between glioblastoma cells is less tight, it might make it easier for immune cells to deeply penetrate the glioblastoma tissue. Second, also intercellular signaling mechanism might be disrupted. It is known that glioblastoma cells build a functional network based on direct cell–cell contacts.^63^ The network architecture is one driver of radioresistance.^63^ Theoretically, this cellular signaling network could also contribute to the failure of immunotherapy and interfering with it might increase chances of therapeutic success. How specific all these potential mechanisms are for immunotherapy will have to be investigated. It may well be that FAK inhibition alone one day is established as a useful therapy augmentation strategy also without concomitant immunotherapy.

Independent of speculative future therapeutic applications, the proteomics and miRNA findings of this research can also lead to clinically useful predictive biomarkers. For instance, only patients with miR-216a or miR-216b above a certain threshold when measured in FFPE samples could be made eligible for DC vaccination immunotherapy. An improved selection and stratification of patients could spare an already vulnerable patient population from undergoing unnecessary procedures (such as leukocytes apheresis). Again, much larger trials with much more patients are required to confirm such biomarker considerations.

In summary, this work establishes feasibility and usefulness of a combined proteomics/miRNomics approach for the investigation of glioblastoma factors related to (immuno)therapy failure. We show that proteomics and miRNomics represent reliable, relevant technologies to identify actionable targets—that we comprehensively mapped here. This paper also introduces the concept of “sensitizing” glioblastoma tissue to immunotherapy. Therewith, we bring a fresh approach to the field of glioblastoma immunotherapy research and support it with first empirical data. Speculatively, further exploring focal adhesion or other factors we identified might help improve glioblastoma care in the future. Other groups can build on the survival factor observations presented here. The proteomics and miRNomics raw data that we made publicly available will be directly useful for that purpose.

## Methods

### General research concept

This work combined mass spectrometry-based quantitative proteomics and miRNA sequencing of tumor tissue from a phase II clinical immunotherapy trial (NCT01213407, EudraCT 2009–015979–27) with the goal of studying survival-relevant factors. The clinical trial (taking place from 2010 to 2015) had investigated a targeted, personalized, autologous, cellular immunotherapy based on tumor lysate-charged DCs (INN Audencel). It failed to show clinical efficacy. For all details regarding the clinical trial, see the recent paper by Buchroithner et al.^19^

For the study of survival-associated factors, principally the same exploratory research concept was used for both, the proteomics and the miRNA analysis. It focused on the identification of discriminatory molecular factors between ST and LT survivors. The respective median of the treatment and the control group was used as the threshold. Where appropriate, a method-specific approach was followed in accordance with the respective requirements of proteomics and miRNomics analyses (see Results section).

### Patient material source

All patients gave their written informed consent regarding the usage of their material for research purposes in the context of the clinical trial. The ethics committee of the Federal State of Upper Austria had approved the study (Number TRX 2/P-II-018). All research was performed in accordance with the relevant local Austrian guidelines and regulations (e.g. AMG, DSG, GSG). For the proteomics analyses, depending on availability, tumor material was used that was originally gained for the production of the cellular immunotherapy in the course of the initial glioblastoma surgery. A part of the material that was later used for charging DCs was hence stored frozen for proteomics research (−80 °C). The miRNA analyses, on the other hand, were based on FFPE material. All available material was fully used up.

### Proteomics analysis (*n* = 36)

Thirty-six patient samples were available for proteomics analyses, of these 16 in the immunotherapy group (prior to Audencel) and 20 in the control group with standard non-immunotherapy treatment. The samples were analyzed by OmicScouts GmbH (Freising, Germany) using a TMT10plex-based quantitative proteomics approach as described earlier.^23^ Batch effects were minimized via normalization as described^23^ and reference samples were well reproduced across all TMT10plex sets. Normalized protein intensities were used for all subsequent data analyses. Bioinformatic analyses were performed in R^64^ and in Perseus.^65^ Protein interactions were obtained from String-DB^66^ v. 10 and visualized in Cytoscape^67^ v. 3.1.1.

The mass spectrometry proteomics data have been deposited to the ProteomeXchange Consortium data repository^68^ with the dataset identifier PXD012616—they are publicly available.

### miRNA analysis (*n* = 38)

Overall, 38 patient samples were available for miRNA analyses, of these 18 in the immunotherapy group (prior to Audencel) and 20 in the control group with standard non-immunotherapy treatment.

First, an exploratory miRNA sequencing study was performed (16 patients). Patients with a pronounced ST or LT outcome were selected and small RNA sequencing was performed. Total RNA was extracted from FFPE samples using the Qiagen miRNeasy kit (Qiagen, Germany). RNA was quantified using the RiboGreen RNA assay, and 200 ng of total RNA was used for library preparation. Ligation of 3′ and 5′ adapters, reverse transcription and PCR amplification (17 cycles) was performed using the NEBNext small RNA Kit. Libraries were pooled using equimolar amounts of miRNA peaks, followed by gel purification of 16–50 nucleotide (nt) fragments. The pool was sequenced on an Illumina NextSeq 500. Read count and miRNA size analysis of all samples exhibited a similar quality and size distribution pattern. Bowtie alignment was done against the following databases: Human Genome, Rfam nrRNA database, Repeated Element database (Repbase), miRbase version 21 and refseq mRNAs. Fifty percent of reads were from mRNA fragments, 20% of reads were unmapped, the rest were valid miRNA reads. Differential expression between ST and LT surviving patients was performed using the “exact” test for two-group comparison by Smyth and Robinson, assuming a negative binomial distribution. Given the exploratory nature of this miRNA analysis phase, *p* values of <0.10 were considered significant.

The next-generation miRNA sequencing data have been deposited to the NCBI Gene Expression Omnibus data repository with the accession number GSE132554—they are publicly available.

Then, after the exploratory phase, a miRNA validation phase followed where an extended number of patients (38 FFPE samples) were analyzed to validate the initial results. For these validation experiments, a selection of miRNAs including miRNAs specific for glioblastoma tumor tissue, the invading tumor margin and “normal” brain tissue was made. In total, this list included 58 different miRNAs. Custom qPCR plates were designed and used to analyze this enlarged set of samples (Supplementary Fig. 4c). Total RNA extraction was performed using the miRNeasy kit (Qiagen, Germany). Reverse transcription was conducted using 2 µl of total RNA diluted to 10 ng/µl concentrations as input for the Exiqon Universal cDNA Kit (Exiqon, Denmark). qPCR amplification was performed using Exiqons ExILENT SYBR® Green Mastermix (Exiqon, Denmark). PCR reactions were performed on a Roche LightCycler 480 II instrument as described previously.^69^ Cq-values were called using the 2nd derivative method.

### Cell culture

To test for sphere formation, glioblastoma cells were cultured in sphere-inducing media^35,36^ in analogy to well-used standard protocols.^70–74^ Briefly, glioblastoma cells were harvested from T75 flasks and brought to T25 flasks with DMEM/F12 (Gibco/ThermoFisher, Waltham, MA, USA) + 20% BIT (Provitro, Berlin, Germany) +20 ng/ml basic fibroblast growth factor (bFGF) + 20 ng/ml epidermal growth factor (EGF; both Stemcell Technologies, Vancouver, Canada). The two patient-derived lines had been established from trial patients.^37^ They are available upon reasonable request. NCH421K is a standard gliomasphere cell line^35,36^ (CSL Cell Lines Service, Eppendorf, Germany). The FAK inhibitor used was 1,2,4,5-benzenetetraamine tetrahydrochloride (Sigma Aldrich, St. Louis, MI, USA).

### The Cancer Genome Atlas

We used the Cancer Genome Browser (genome-cancer.ucsc.edu) made available by the University of California San Francisco and accessed the two largest datasets: one by the Broad Institute of MIT/Harvard (Affymetrix gene expression measurement) and one by the University of North Carolina (Agilent gene expression measurement).

### Statistical analyses

Specific statistical methods for the proteomics or miRNomics analysis are mentioned in the respective paragraphs. General statistical framework: differences between two groups were analyzed via Student's *t*-test (two-sided). For the analysis of differences between multiple groups (e.g. for the tissue-specific miRNA profiles for glioblastoma, invading tumor margin and “normal” brain tissue), one-way ANOVA was used. Multiple testing corrections were applied as described in the respective method section. Clinical relevance of a marker was assessed via generating Kaplan–Meier curves and performing log-rank testing. Unless otherwise stated, *p* values of <0.05 were considered significant. Software used included Microsoft Excel, GraphPad Prism, Qlucore Omics Explorer and RStudio.

### Reporting summary

Further information on research design is available in the Nature Research Reporting Summary linked to this article.

## Supplementary information

Supplementary Material

Reporting Summary

## Data Availability

The data reported in this study are available from the data repositories ProteomeXchange Consortium and NCBI Gene Expression Omnibus (see above).
