# Supplementary material for: Combined proteomics/miRNomics of dendritic cell immunotherapy-treated glioblastoma patients as a screening for survival-associated factors
Source: NPJ Vaccines. 2020 Jan 16;5:5. doi: 10.1038/s41541-019-0149-x (PMC6965118; doi:10.1038/s41541-019-0149-x)

Combined proteomics/miRNomics of dendritic cell immunotherapy-treated glioblastoma patients as a screening for survival-associated factors

SUPPLEMENTARY MATERIAL

Friedrich Erhart*, Matthias Hackl, Hannes Hahne, Johanna Buchroithner, Chen Meng, Simone Klingenbrunner, René Reitermaier, Katrin Fischhuber, Susanna Skalicky, Walter Berger, Sabine Spiegl-Kreinecker, Daniela Lötsch, Gerda Ricken, Bernhard Kuster, Adelheid Wöhrer, Georg Widhalm, Johannes Hainfellner, Thomas Felzmann, Alexander M. Dohnal, Christine Marosi and Carmen Visus

**Corresponding Author:* Dr. Friedrich Erhart, Department of Neurosurgery, Medical University of Vienna, Währinger Gürtel 18-20, 1090 Vienna, friedrich.erhart@meduniwien.ac.at, +43/650/2718182

Supplementary Figure S1. Estimation of protein amount in the initial feasibility phase (n=2 patients, error bars=SEM), which was done as the first step of the proteomics analysis to get an impression on the quantitative protein amount available in the samples, i.e. whether it would be sufficient for analyses. Three different sources of material for proteomics measurement were tested: (i) the tumor protein lysate that was also used for charging DCs, (ii) tumor single cell suspensions and (iii) dry pellets that had been generated from the single cell suspensions. **(a)** The provided sample type “lysate” had sufficient protein content for further analysis and it was thus chosen. **(b)** Illustrative proteome profiling of one ST and one LT sample.

a

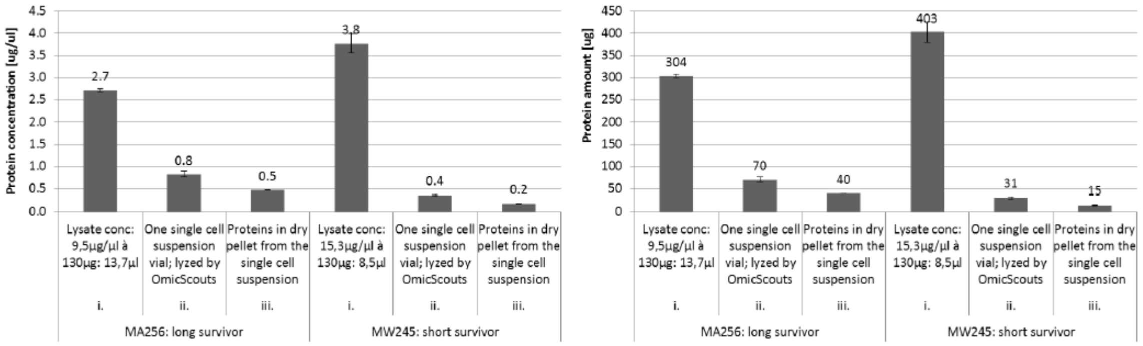

b

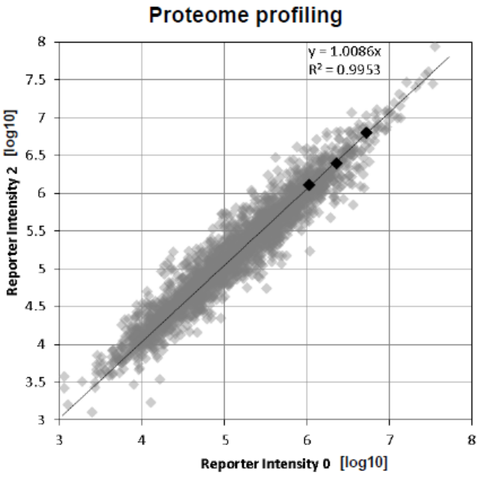

Supplementary Figure S2. Depiction of findings from the protein measurement feasibility pilot phase. **(a)** Pilot proteomics of the feasibility samples detected proteins overexpressed in an ST (“MA256”) and an LT (“MW245”) immunotherapy patient. For the generation of the graph, the measured (protein) intensities were mapped along the relative frequency in the two respective samples (x-axis), which visualizes the proteins specifically overexpressed (red labelling) in the respective sample (i.e. “MA256” or “MW245”). Then the respective gene ontology (GO) terms were added as a clarification. **(b)** In the LT patient, among the molecules that were enriched, was also the focal adhesion kinase 1 (FAK1).

a

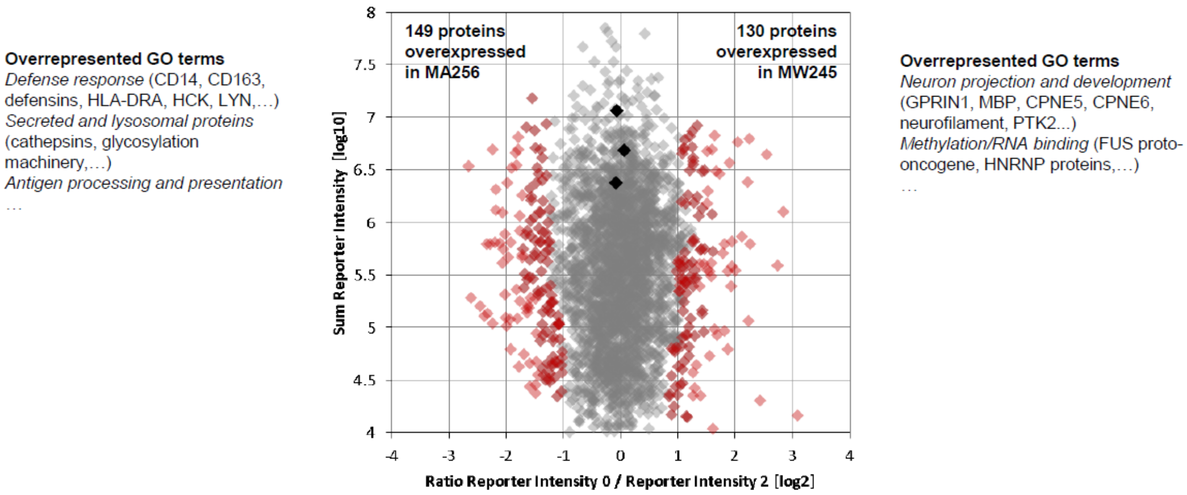

b

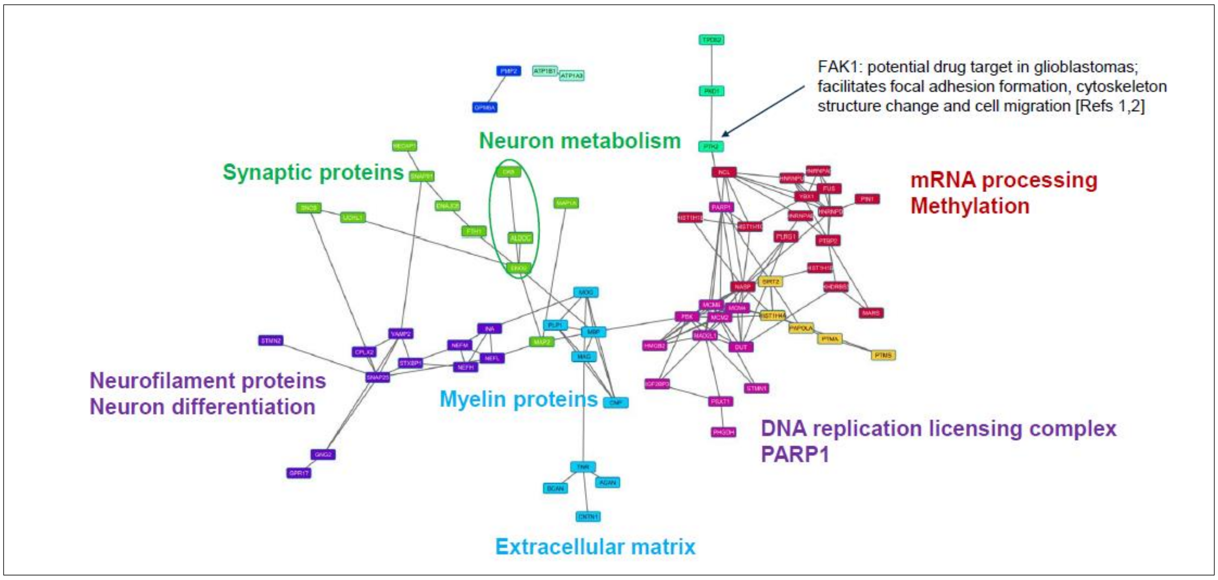

Supplementary Figure S3. Evaluation of a potential impact of known factors with a survival association in the proteomics immunotherapy specimen set (n=16 patients analyzed overall with specific n depending on information availability: e.g. MGMT was not measured in all study patients). Note: For extent-of-resection (EOR), the stratification into groups followed the group definition also used in Buchroithner et al, Cancers, 2018). None of the factors measured had a significant survival-association in Kaplan-Meier analyses. For MGMT a tendency is visually detectable but not significant.

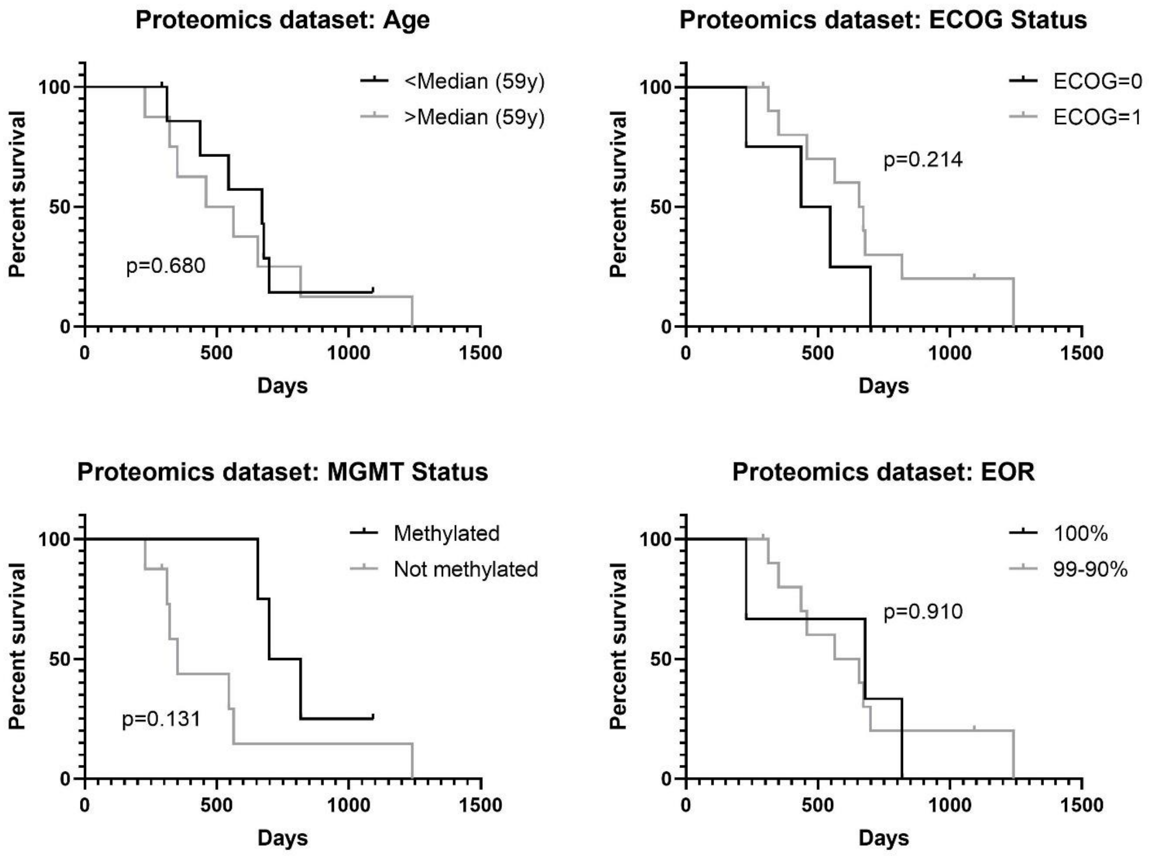

Supplementary Figure S4. Results of a principal component (PC) analysis that synthesizes the information in a dataset in a way that the major determining principal components are identified. Via analyzing the distribution of the processed sample *batches* – since sample processing was performed in batches due to the technical requirements of the measurement system – with regards to the identified principal components, it can be evaluated if the batches cluster in a certain quadrant. This would then indicate batch effects. In the case of the dataset evaluated in the context of the present study, no batch effects of the samples measured were detectable (n=36).

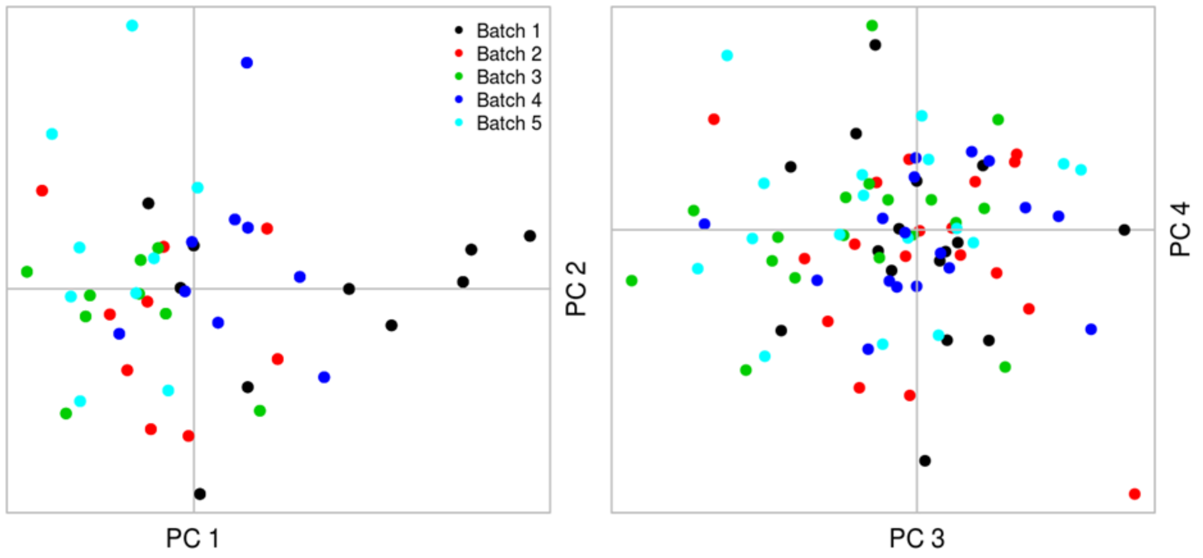

Supplementary Figure S5. Results of K-means clustering (5 clusters) and principal component analysis (PCA) of the samples in the *clusters* (and not the batches, see Figure S4) and the respective identification of overrepresented proteins (n=36 patients available for proteomics analysis). Generally, the proteins present in the measured samples comprised protein sets like synaptic proteins (cluster 3), oxygen transport and other blood proteins (cluster 1) and antigen processing proteins (cluster 5 samples). Cluster 2 represents the pooled reference samples.

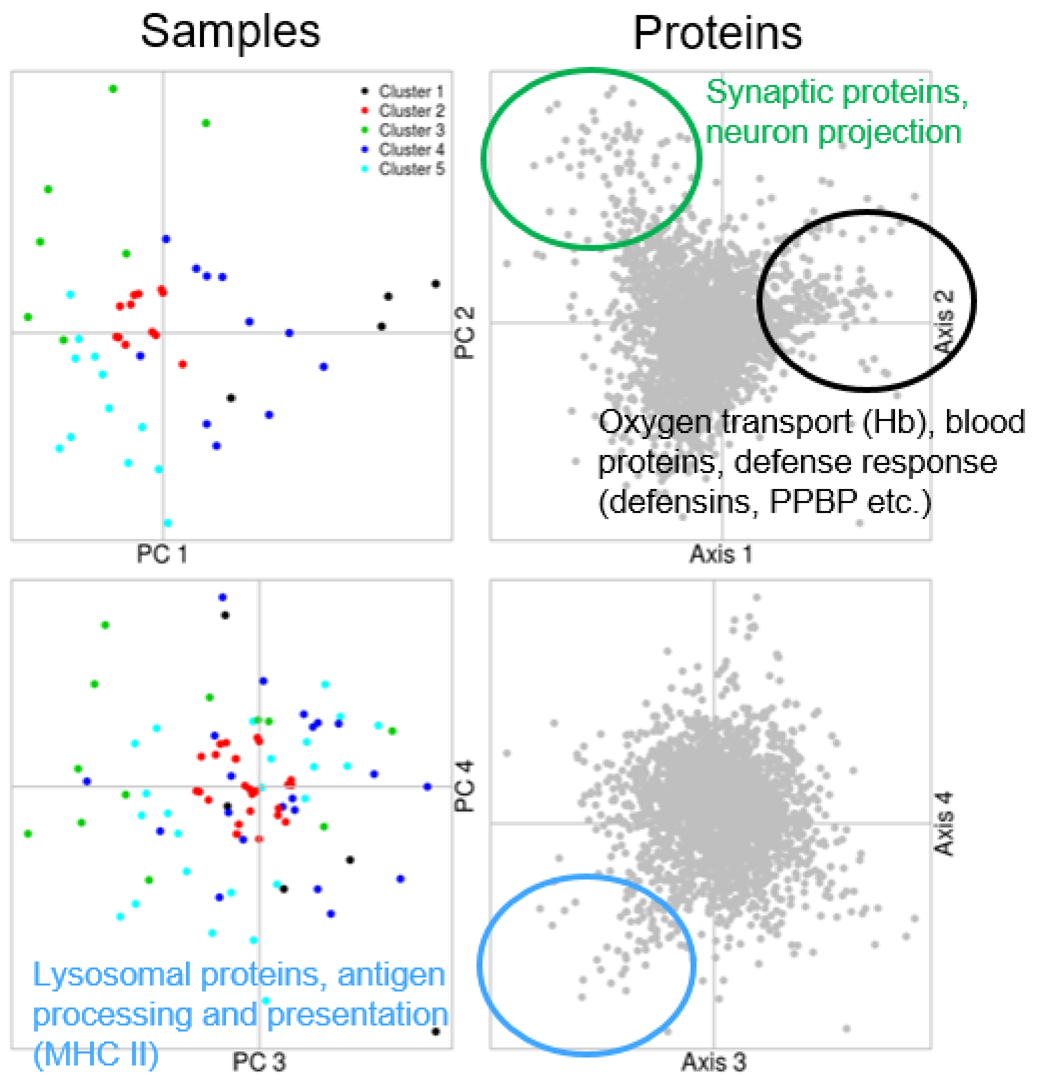

Supplementary Figure S6. In the control group with standard non-immunotherapy glioblastoma treatment (n=20 patients with sample available for proteomics), ST surviving patients (**a**) and LT surviving (**b**) patients had a distinct proteome, that was mapped here as an illustration. The maps organize the identified proteins in a way that their affiliation to specific biological pathways and mechanisms is highlighted.

a Short-term (ST) survivors

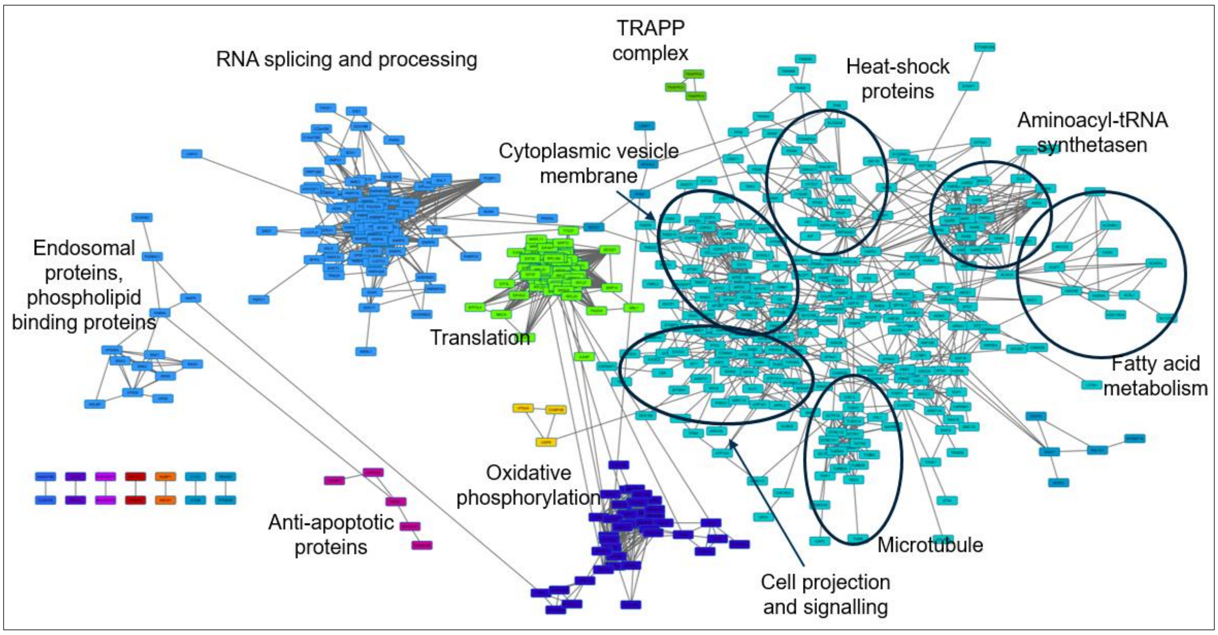

b Long-term (LT) survivors

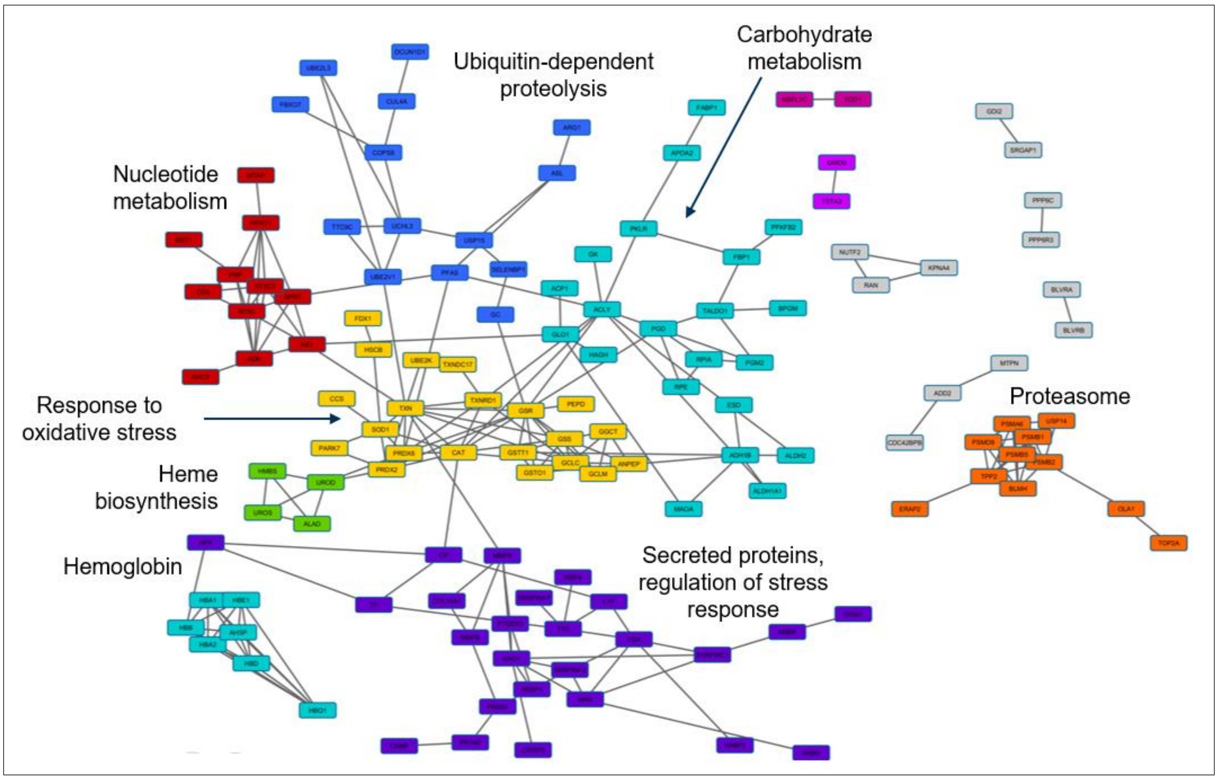

Supplementary Table S1. List of protein kinases with possible roles as cancer drivers and/or resistance factors identified by quantitative proteomics in the control group patient samples.

Gene name	Protein name
CAMK2D	calcium/calmodulin-dependent protein kinase II delta
CDK5	cyclin-dependent kinase 5
CSK	c-src tyrosine kinase
CSNK2A1	Catalytic subunit of a constitutively active serine/threonine-protein kinase complex
GSK3B	glycogen synthase kinase 3 beta
MAPK1	mitogen-activated protein kinase 1
MAPK15	mitogen-activated protein kinase 15
MAPK3	mitogen-activated protein kinase 3
AMPK (PRKAA1, PRKAG1)	AMP-activated protein kinase
PKC	protein kinase C
PRKRA	Interferon-inducible double-stranded RNA-dependent protein kinase activator A
PTK2	PTK2 protein tyrosine kinase 2 = FAK1
PTK2B	PTK2B protein tyrosine kinase 2 beta = FAK2
STK4	serine/threonine kinase 4
TAOK1	TAO kinase 1
TBK1	TANK-binding kinase 1

Supplementary Table S2. Consensus list of possible resistance factors identified in the immunotherapy treatment group patients based on a combination of supervised clustering and elastic net analysis.

**Protein name**
HIP1
IGF2R
RBP1
C9orf64
EPDR1, UCC1
FRYL
FTH1
SDCBP
ADPRHL2
FABP5
SDF4
DPP6
MAP1LC3A
POFUT2
GATM
WDR82
AKT1

Supplementary Figure S7. Evaluation of the role that proteins identified as part of the immunotherapy group analysis could have also in the (standard-of-care, SOC) control group (n=20). Of the 6 proteins investigated here, 2 were also significant in the control group (HIP1 and IGF2R).

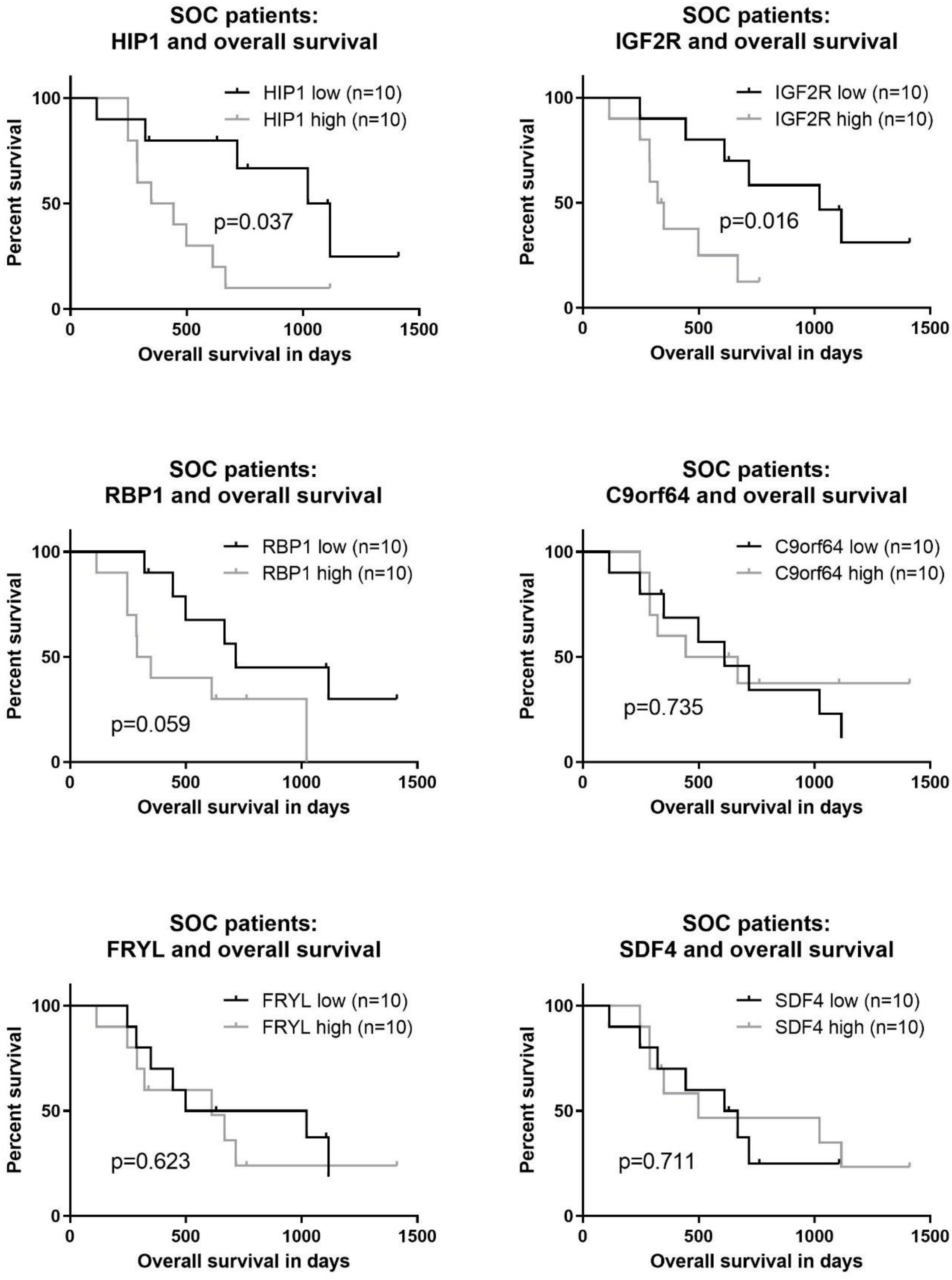

Supplementary Figure S8. Evaluation of a potential impact of known factors with a survival association in the miRNomics immunotherapy specimen set (n=18 patients analyzed overall with specific n depending on information availability: e.g. MGMT was not measured in all study patients). For extent-of-resection (EOR), the stratification into groups followed the group definition also used in Buchroithner et al, Cancers, 2018). Again, none of the factors measured had a significant survival-association in Kaplan-Meier analyses. And again, for MGMT a tendency is visually detectable but not significant.

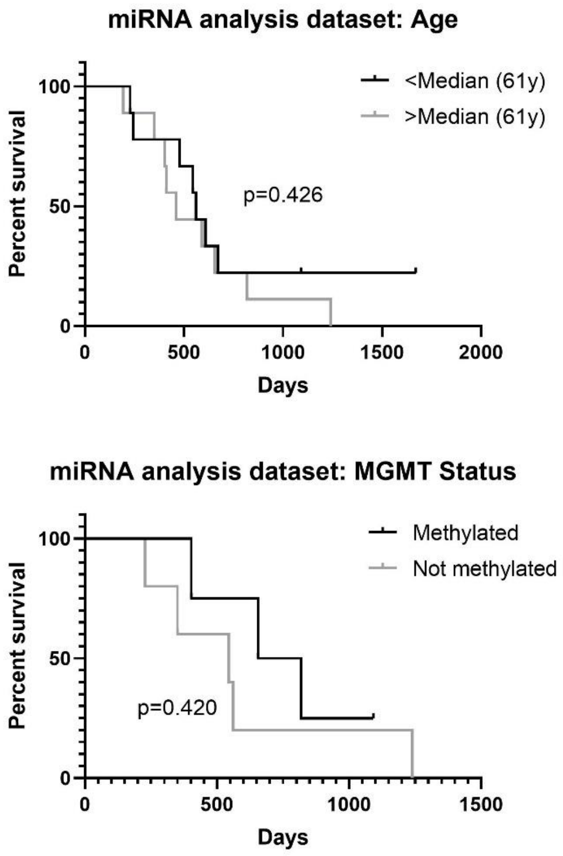

Supplementary Figure S9. Exemplary depiction of quality control characteristics of one miRNA measurement sample. (**a**) Base quality. (**b**) Size distribution. (**c**) GC content distribution.

a

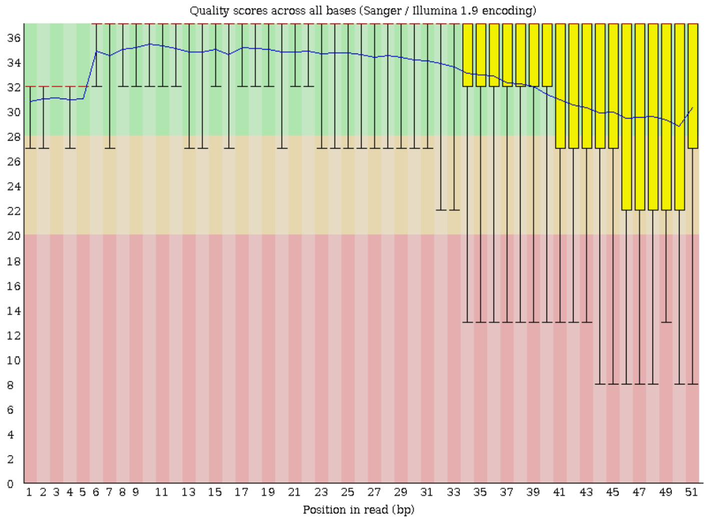

b

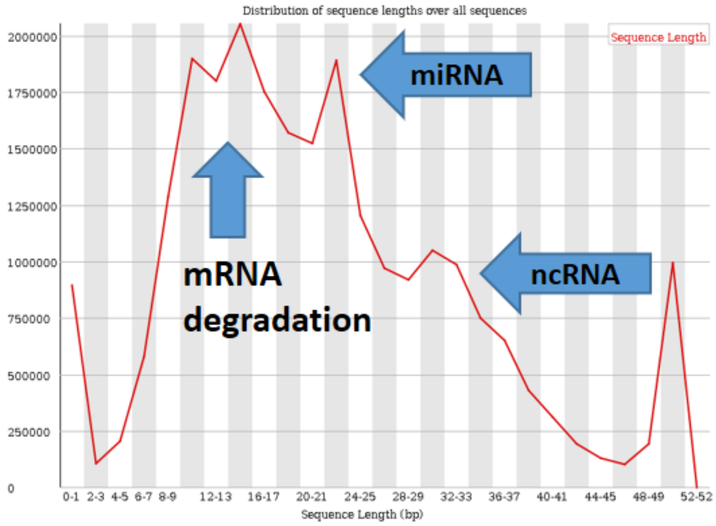

c

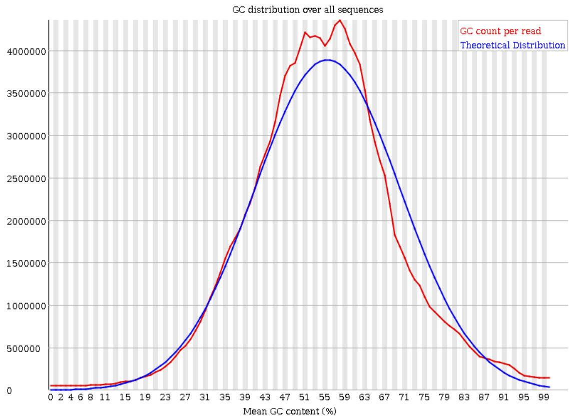

Supplementary Table S3. List of all miRNAs that were part of the qRT-PCR validation panel.

hsa-miR-216b-5p
hsa-miR-216a-5p
hsa-miR-708-3p
hsa-let-7i-3p
hsa-miR-200a-3p
hsa-miR-887-3p
hsa-miR-30a-3p
hsa-miR-217
hsa-miR-708-5p
hsa-miR-195-3p
hsa-miR-30a-5p
hsa-miR-146b-5p
hsa-miR-99a-3p
hsa-miR-29c-5p
hsa-miR-455-3p
hsa-miR-93-5p
hsa-miR-455-5p
hsa-miR-146b-3p
hsa-miR-21-5p
hsa-miR-21-3p
hsa-miR-338-3p
hsa-let-7d-3p
hsa-miR-339-3p
hsa-miR-182-5p
hsa-miR-183-5p
hsa-miR-186-5p
hsa-miR-26a-5p
hsa-miR-212-3p
hsa-miR-1260a
hsa-miR-485-5p
hsa-miR-95-3p
hsa-miR-219a-2-3p
hsa-miR-1
hsa-miR-136-3p
hsa-miR-382-5p
hsa-miR-210-3p
hsa-miR-409-3p
hsa-miR-543
hsa-miR-654-3p
hsa-miR-135a-5p
hsa-miR-411-5p
hsa-miR-885-3p
hsa-miR-654-5p
hsa-miR-370-3p

hsa-miR-204-5p
hsa-miR-409-5p
hsa-miR-485-3p
hsa-miR-625-3p
hsa-miR-377-3p
hsa-miR-199b-5p
hsa-miR-299-5p
hsa-miR-196a-5p
hsa-miR-487a-3p
hsa-miR-382-3p
hsa-miR-889-3p
hsa-miR-10b-5p
hsa-miR-494-3p
hsa-miR-615-3p

Supplementary Figure S10. Correlation of miR-216b and FAK in the samples where both proteomics and miRNA data was available (n=22). A trend towards a negative correlation is seen but does not reach statistical significance.

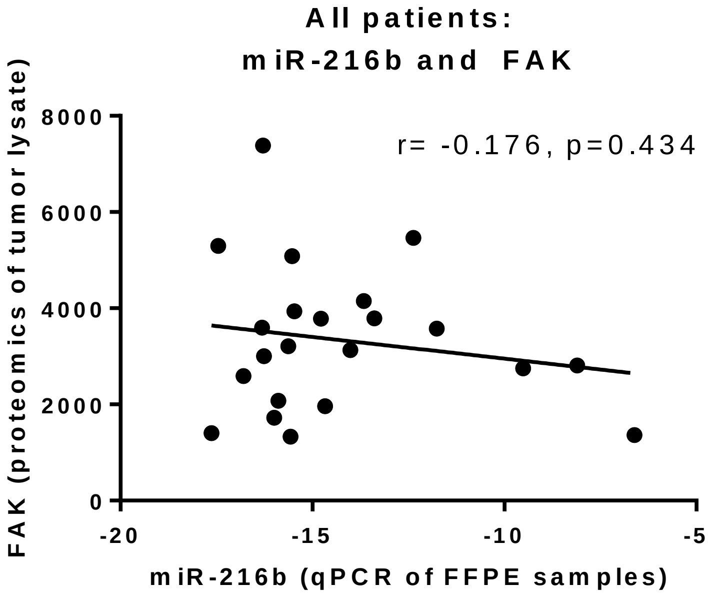

Supplement: Supplementary file 1 — Supplementary Material [file 41541_2019_149_MOESM1_ESM.pdf]
